# Epicardial adipocytes in the pathogenesis of atrial fibrillation: An update on basic and translational studies

**DOI:** 10.3389/fendo.2023.1154824

**Published:** 2023-03-20

**Authors:** Brooks Willar, Khan-Van Tran, Timothy P. Fitzgibbons

**Affiliations:** Department of Medicine, Cardiovascular Division, University of Massachusetts Chan Medical School, Worcester, MA, United States

**Keywords:** epicardial adipose tissue, obesity, atrial fibrillation, inflammation, left atrium, remodeling, adipocyte

## Abstract

Epicardial adipose tissue (EAT) is an endocrine organ containing a host of cell types and undoubtedly serving a multitude of important physiologic functions. Aging and obesity cause hypertrophy of EAT. There is great interest in the possible connection between EAT and cardiovascular disease, in particular, atrial fibrillation (AF). Increased EAT is independently associated with AF and adverse events after AF ablation (e.g., recurrence of AF, and stroke). In general, the amount of EAT correlates with BMI or visceral adiposity. Yet on a molecular level, there are similarities and differences between epicardial and abdominal visceral adipocytes. In comparison to subcutaneous adipose tissue, both depots are enriched in inflammatory cells and chemokines, even in normal conditions. On the other hand, in comparison to visceral fat, epicardial adipocytes have an increased rate of fatty acid release, decreased size, and increased vascularity. Several studies have described an association between fibrosis of EAT and fibrosis of the underlying atrial myocardium. Others have discovered paracrine factors released from EAT that could possibly mediate this association. In addition to the adjacent atrial cardiomyocytes, EAT contains a robust stromal-vascular fraction and surrounds the ganglionic plexi of the cardiac autonomic nervous system (cANS). The importance of the cANS in the pathogenesis of atrial fibrillation is well known, and it is quite likely that there is feedback between EAT and the cANS. This complex interplay may be crucial to the maintenance of normal sinus rhythm or the development of atrial fibrillation. The extent the adipocyte is a microcosm of metabolic health in the individual patient may determine which is the predominant rhythm.

## Introduction

**Figure 1:**
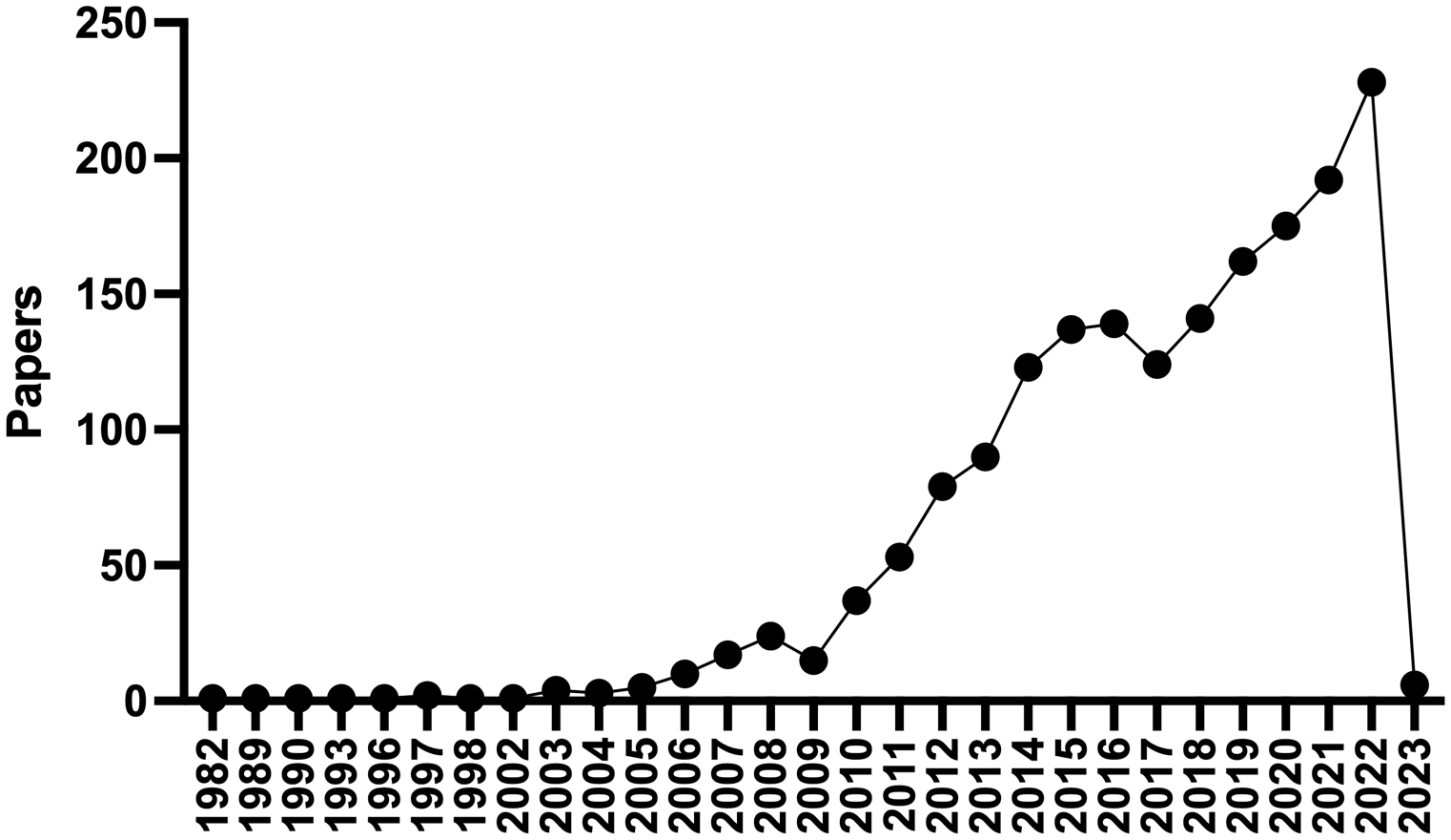
PubMed Timeline Results for “Epicardial adipose tissue”. There has been a dramatic increase in the number of publications regarding epicardial adipose tissue since 2003.

Atrial fibrillation (AF) is the most common sustained arrhythmia in adults, affecting 37% of patients over the age of 55, hence the importance of risk factor identification and modification (1). Environmental factors including chronic disease, age, and acute triggers have been implicated as risk factors for developing AF. Coronary artery disease (CAD), hypertension, obesity, diabetes, obstructive sleep apnea (2), chronic kidney disease (CKD), and inflammatory diseases increase the risk for AF as do acute triggers such as binge drinking and physical stressors (e.g., infection, surgery, and metabolic derangements). AF has been associated with an increased incidence of stroke, heart failure, dementia, as well as death and globally, AF is associated with an increased risk for mortality and morbidity and accounted for 6 million disability adjusted life years in 2017 (1, 3). Given this impact upon human health there is a great need for interventions to prevent the development of AF and treat prevalent cases. Epicardial adipose tissue (EAT) has garnered significant interest as a direct connection between obesity and cardiovascular disease, and AF in particular. To the extent that EAT may be modified by behavioral, pharmacologic, or even surgical interventions it is an attractive therapeutic target to combat AF. A PUBMED search for “epicardial adipose tissue” returns 1,509 results, with 228 in 2022 alone (**Figure 1**). Since 2003, commensurate with the publication of landmark studies by Mazurek and Iacobellis, there has been a burgeoning interest in the effects of EAT on cardiovascular pathophysiology (4, 5). There have been numerous recent comprehensive reviews on this topic to which the reader is referred (6–9). Herein we will aim to focus more on the biology of epicardial adipocytes and how this might affect neighboring cardiomyocytes and contribute to the pathogenesis of AF. Our goal is to stimulate the readers interest and perhaps spark new lines of inquiry in this fascinating and important field.

## Pathophysiology of atrial fibrillation

The mechanism for developing AF is not clearly understood but likely changes as a patient progresses from paroxysmal AF to long-standing persistent AF. With this progression the impetus transitions from an arrhythmogenic trigger to an electro pathology mediated trigger (3). Paroxysmal AF, an arrhythmia that self-terminates within 7 days, is typically driven by the cardiac muscle sleeve around the pulmonary veins (PVs) in 90% of cases and is associated with rapid focal activity and local reentry. As a patient transitions to persistent AF, requiring pharmacological or electrical cardioversion, the driver evolves into electrical remodeling of ion channels and irreversible structural changes to the atria (10). We hypothesize that any primary effect of epicardial adipocytes would be in the paroxysmal stage. However, to the extent that cross talk between atrial myocytes, epicardial adipocytes, and the cardiac autonomic nervous system (cANS) can occur, adipocytes may play other roles in the persistent/permanent stage of AF, by modulating the cANs afferent signals to the brain and promoting efferent cANs discharge (11).

### Molecular changes in atrial myocytes

In terms of the primary changes in the atrial myocardium, electrical remodeling from altered expression and functioning of cardiac ion channels favors the development of functional reentry substrates (10). The molecular mechanism leading to repolarization changes are not clearly understood, but are thought to involve sodium, potassium, and calcium channels. Atrial myocytes in patients with AF show unchanged or slightly reduced sodium current amplitude. Reduced calcium channel density is consistent in patients with AF and may be a determinant of shortening refractoriness and arrhythmogenesis. In addition, the sarcoplasmic reticulum’s handling of calcium, which is affected by alterations in ryanodine receptor channels (RyR2), can lead to calcium leak and thus modulation of RyR2, which is a prevalent finding in this patient population. Patients with AF have a more negative resting membrane potential indicating that potassium channels may also play a role in the development of AF. Remodeling of gap junction subunits such as connexin may also be involved in a genetic predisposition to AF (12).

### Fibrosis, fibrofatty infiltration, and ganglionic plexi

**Figure 2:**
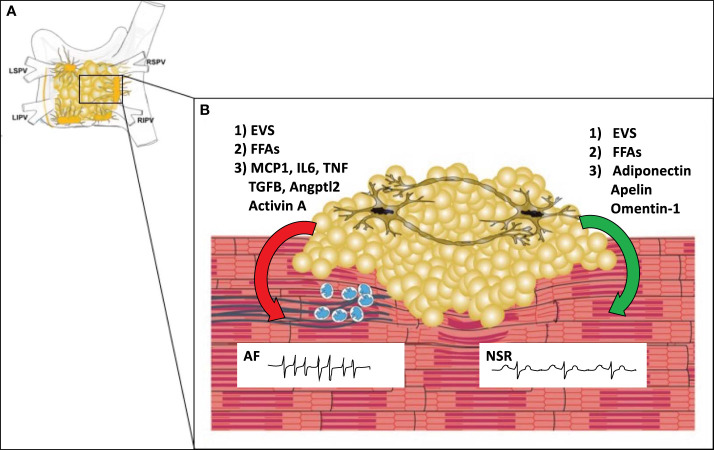
The Microenvironment of the Posterior Left Atrial Wall. **(A)** The posterior left atrial wall has variable amounts of EAT, which includes the four major ganglionic plexi and confluence of the pulmonary veins. **(B)** EAT covers portions of the left atrial wall and includes the ganglionic plexi. In obesity (left), EAT may promote infiltration of macrophages (blue) *via* secretion of MCP1 and other pro-inflammatory cytokines. Macrophages and or myofibroblasts then promote atrial fibrosis (blue lines) with stimulation of pro-fibrotic factors such as TGFB, Angptl2, and Activin A. This causes fibrosis of the atrial myocardium which creates a substrate for reentry leading to the development of atrial fibrillation. In normal conditions (right), EAT secretes anti-inflammatory and anti-fibrotic paracrine factors (adiponectin, apelin, omentin-1) that help maintain normal electrophysiologic properties of the atrial myocardium leading to normal sinus rhythm (NSR). In both obese and normal conditions epicardial adipocytes may secrete other factors such as extracellular vesicles (EVS) or free fatty acids (FFAs) which may have protective or adverse effects depending upon the EV contents and particular fatty acid species. MCP1 (macrophage chemoattractant protein-1), IL6 (Interleukin 6), TGFB (transforming growth factor beta), TNF (tumor necrosis factor), Angptl2 (Angiopoietin Like 2), LSPV (left superior pulmonary vein), LIPV (left inferior pulmonary vein), RSPV (right superior pulmonary vein), RIPV (right inferior pulmonary vein).

Structural changes, such as fibrosis, neural/autonomic remodeling, and anatomic features also contribute to the pathogenesis of AF. Fibrosis can separate muscle bundles, replace dead myocytes, and can couple electrically to cardiomyocytes, leading to reentry and ectopic activity. Fibrosis leads to progression from paroxysmal to permanent forms of AF, which in turn creates a positive feedback loop of increased fibrosis. In addition to fibrosis, infiltration of the atrial myocardium by epicardial fat can contribute to atrial conduction abnormalities (13). Autonomic/neural remodeling through vagal discharge, beta-adrenoceptor activation, and atrial sympathetic hyper-innervation can also contribute to positive feedback loops that promote AF persistence and recurrence. The left and right atria including PVs, LA posterior wall/roof, the ligament of Marshall, as well as the vena cava have features that promote both focal and reentrant triggers (10). The junction of the pulmonary veins and left atrium has a constellation of ganglionic plexi that receive input from both the sympathetic and parasympathetic autonomic nervous system (**Figure 2**) (11). It is estimated that each plexus contains 200-1000 neurons. AF can be induced by activation of these GPs. On the other hand, ablation of these plexi and the ostia of the pulmonary veins can cure or reduce arrythmia burden in patients with AF (11). The interaction of epicardial adipocytes with the GPs of the cANS has yet to be investigated. However, it is well established that other adipose depots receive important input from the autonomic nervous system, and feed back to the ANS to provide metabolic signals to the CNS (14, 15). Recent studies have shown that macrophages associated with sympathetic neurons (SAMs) help to regulate this interaction. For example, in obesity, SAMs in brown adipose tissue induce expression of solute carrier family 6 member 2 (*SLC6A2*) and monoamine oxidase A(*MAOA*) (2). SLC6A2 is a membrane transporter for norepinephrine (NE) and MAOA is an enzyme responsible for enzymatic degradation of NE. With obesity, the induction of these proteins in SAMS causes a reduction in the sympathetic activation of brown adipose tissue, thus reducing thermogenesis and energy expenditure (2). We hypothesize that macrophages in EAT may mediate interactions with neurons in cardiac GPs in a similar fashion, allowing for changes in metabolism to regulate afferent feedback from the cANs to the brain.

### Left atrial size and obesity

On a macroscopic scale, left atrial size, inflammation, and obesity all seem to play important roles in the development of AF and the progression from paroxysmal to permanent subtypes. Patients with paroxysmal AF have smaller LA diameter (4.3 vs 4.8cm) and fewer incidences of LA >5cm as compared to patients with permanent AF (16). This is particularly important as LA enlargement is a significant predictor of stroke in men and death in both sexes (17). Regarding obesity, there is a strong association between obesity (BMI >30) and AF. The Framingham heart study showed that for every 1 unit of increased BMI above 25, there was a 4% increase in AF risk. This conclusion was supported by a large meta-analysis showing that for every 5 unit increase in BMI, AF incidence increased by 29% (18). The mechanism of obesity increasing the incidence of AF may be related to epicardial and abdominal adiposity. A large meta-analysis by Wong showed that epicardial fat, waist circumference, and waist to hip ratio were all associated with a higher incidence of AF. However, the strength of association was highest for epicardial fat (19). Epicardial fat, given its proximity to the myocardial cells may confer structural remodeling like the substrates described above.

## Epicardial adipocytes; the same, but different

It is now well understood that adipose tissue is an active organ vital to human health (20). Any given adipose depot is comprised of a multitude of cell types, and it is easiest to consider it in terms of the adipocyte and stromal vascular fraction (SVF). The SVF is comprised of all the non-adipocyte cells within a certain fat pad. These may include, but are not limited to, fibroblasts, stem cells, neurons, vascular cells, and a broad array of immune cells. Broadly speaking, there are three different types of adipocytes; white, beige and brown (20). Brown adipocytes are adipocytes that generate heat by uncoupling the respiratory chain *via* the protein uncoupling protein 1 (UCP1). Brown adipose is present in babies and in smaller quantities in metabolically healthy adults. It is activated by the sympathetic nervous system in response to cold exposure, by NE binding to the β3 adrenergic receptor. Brown adipocytes express UCP1 constitutively and expression is reduced in chronic thermoneutral conditions (20). In contrast, beige adipocytes are adipocytes within subcutaneous fat that induce UCP1 expression rapidly upon cold exposure. Beige and brown adipocytes generate heat and contribute to metabolic health by increasing energy expenditure and releasing “BATokines” which have salutary effects of peripheral tissues. The amount of brown and beige adipocytes decreases with aging and obesity (20).

In contrast to brown adipocytes, the principal role of white adipocytes is to sequester lipid by lipogenesis or regulating lipolysis. They also secrete bioactive molecules (e.g., extracellular vesicles, lipokines, and adipokines) that target systemic organs. The mean volume of a white adipocyte is proportional to the rate of lipogenesis, the rate of lipolysis, and the nutrient supply/blood flow (21). EAT and visceral adipose are both predominantly white adipose depots and similar in many respects. Generally, EAT volume correlates with visceral adiposity, as measured by echocardiogram and CT. Epicardial adipocytes and adipocytes from visceral fat depots are smaller than subcutaneous adipocytes (22). EAT has a greater concentration of capillaries and increased expression of a broad array of inflammatory markers (21, 22). On the other hand, there are subtle differences between epicardial and adipocytes from fat within the abdominal cavity (21). Ovine epicardial adipocytes have a greater ratio of oleic acid (18:1) to stearic acid (18:0) than peri-renal or omental adipocytes. Although stearoyl-CoA desaturase (SCD) expression was lowest in epicardial fat compared to all other depots, there was a high correlation between SCD expression and oleic acid content in epicardial adipocytes, whereas there was no correlation in visceral adipocytes (21). Like visceral adipose tissue, EAT has a greater expression of inflammatory genes than subcutaneous adipose tissue (22, 23). This is true even in the absence of cardiovascular disease or obesity. In humans with obesity and in animal models of high fat feeding visceral adipose becomes inflamed which leads to dysfunctional adipocyte biology, lipotoxicity, and insulin resistance in remote tissues. This vast field of research (immunometabolism) has ushered in a new era of discovery, investigation, and therapeutic opportunity that is beyond the scope of this review (20, 24). However, whether epicardial adipose tissue responds in the same way to high fat feeding/obesity has yet to be shown. White perivascular adipose tissue surrounding the abdominal aorta in mice becomes severely inflamed with HFD (25). Given the similarities between VAT and EAT, it is reasonable to consider the possibility that epicardial fat does too.

The predominant stimulus for adipose inflammation in response to obesity remains elusive. Adipocyte hypertrophy is likely a primary event, followed by immune cell infiltration, apoptosis, revascularization, and fibrosis (9, 26). In obese conditions macrophages account for 40-50% of cells in visceral adipose tissue (27). There are many different populations of macrophages in adipose tissue. Adipose tissue resident macrophages are present even in lean conditions to help maintain tissue homeostasis. With the onset of obesity and adipocyte hypertrophy, chemokines such as monocyte chemoattractant protein-1 (MCP-1) stimulate infiltration of monocyte derived macrophages (CD11b+, CD11c+, F4/80+). These macrophages are pro-inflammatory and express factors such as TNFα, IL-1β, IL6 and NO (26). Thus, a chronic inflammatory state is established in visceral adipose tissue, leading to failure to effective store TG, lipotoxicity, and subsequent insulin resistance in skeletal muscle and liver. Whether or not these processes occur in EAT has yet to be established.

## Basic and translational studies of EAT

For purposes of this section, EAT will be considered to be adjacent to the atrial myocardium of interest unless noted otherwise. It should be mentioned that the amount of EAT adjacent to the right atrium, left atrium and pulmonary veins is highly variable. Studying EAT in small animal models is problematic, because rats and mice do not have epicardial adipose, except after prolonged high fat diet (28). Furthermore, AF is not common in mice except in only a few genetic strains. Therefore, atrial fibrillation and EAT are both usually studied in large animal models (i.e., sheep, dogs, or rabbits), often using the artificial rapid atrial pacing(RAP) (18) model to induce AF.

Li et al. studied the effect of RAP on EAT. 6 weeks of RAP induced AF and lowered the effective refractory periods in the left and right atria (29). This was associated with increased reactive oxygenated species (ROS) production and phosphorylation of NF-κB. Concentrations of inflammatory cytokines such as TNFα, IL6, and TGFβ were increased in the left atrial myocytes and EAT (29). Histology showed atrial and adipose tissue fibrosis, in addition to adipocyte infiltration into the atrial myocardium. PPARγ and Adiponectin expression was reduced in EAT. Metformin reversed these alterations. These findings are analogous to prior studies of perivascular fat in high fat diet fed mice; obese mice had reduced Adiponectin expression in perivascular fat surrounding the femoral artery (30). Loss of adiponectin resulted in decreased nitric oxide synthase activation and vasoconstriction. With the onset of obesity expression of beneficial paracrine factors in perivascular adipocytes is lost; this may also be true of aging adipocytes. In addition to the loss of beneficial factors, expression of pathologic paracrine factors is increased, for example TNFα, which promotes neointimal hyperplasia (31). Therefore, it seems that like perivascular adipocytes, peri-atrial epicardial adipocytes may signal *via* paracrine mechanisms to the underlying atrial cardiomyocytes. In this case, loss of adiponectin may result in reduced SERCA2a expression and abnormal calcium handling. Many other studies have shown putative adverse effects of factors secreted by EAT (32–35).

Venteclef et al. showed that the EAT secretome stimulated fibrosis in rat atria *in vitro*. This fibrosis was associated with associated with high Activin A concentrations in EAT; in contrast SAT had low Activin A concentrations (32). The fibrotic effects of Activin A *in vitro* were blocked with an antibody targeting activin A.

Nalliah et al. conducted an elegant study of the right atrial appendage of humans without AF who were having heart surgery (13). Greater amounts of EAT around the right atrial appendage correlated with slow conduction, electrogram fractionation, and fibrosis of the underlying atrial myocardium. It was also noted that the gap junction protein connexin-40 migrated laterally in subjects with increased EAT, becoming dissociated from cadherin (13). Conditioned media from sheep EAT altered the electrophysiologic properties of human induced pluripotent stem cell derived cardiomyocytes (hiPSC-CMS), resulting in a decreased spontaneous beating rate and prolonged field potential duration in comparison to non-conditioned media. Proteomic analysis of murine pericardial fat and inguinal fat was then performed. Pericardial fat was enriched in proteins that regulate cell metabolism (e.g., ATP-citate synthase, alcohol dehydrogenase class 3, Long-chain enoyl-COA hydratase). The top enriched cellular component pathway was “focal adhesion” (GO:0005925). Interestingly, the top two GO biological processes were “fatty acid beta oxidation” (GO:0006635) and “cellular response to interleukin12” (GO:0070671) (13). It should be noted that this proteomic analysis was done using secreted proteins from murine pericardial fat, which is likely quite different from EAT (36).

Abe et al. studied the resected left atrial appendages and associated EAT from 59 consecutive cardiac surgery patients with AF (35). Fibrosis of EAT was associated with left atrial fibrosis. The collagen content of left atrial myocardium correlated with inflammatory proteins in EAT (TNFα, MCP-1, IL6, VEGF, MMP2, and MMP9). Expression of HIF-1α and Angptl2 was associated with inflammation in EAT. In a second study, the same group showed that treatment of rat atria *in vitro* with Angptl2 caused fibrosis; this effect was reversed with an anti-Angptl2 antibody (33). Angptl2 caused an increase in expression of α-smooth muscle actin, TGFβ1, and stimulated phosphorylation of ERK, inhibitor of κBa, and p38 MAPK. The authors concluded that antagonism of Angptl2 in EAT may be a therapeutic option for the prevention of AF (33).

In addition to aging and obesity/insulin resistance, a third mechanism that may contribute to EAT inflammation is hypoxia. Obstructive sleep apnea (2) is a known risk factor for the development of AF and untreated OSA leads to recurrence after AF ablation or cardioversion (37–39). In canine models of OSA induced AF there is evidence of cANS hyperactivity in ganglionic plexi of the left atrium (40). The ganglionic plexi are surrounded by EAT and form a ring around the confluence of the pulmonary veins (**Figure 2**). Dai et al. studied the effects of chronic OSA on EAT in canines (41). Chronic OSA resulted in a dramatic fibrosis of EAT and the adjacent atrial myocardium. OSA resulted in increased expression of inflammatory markers in EAT, including Activin A, TGFβ1, MMP9, TNFα, and IL-6. Treatment with metoprolol reversed fibrosis and lowered inflammatory marker expression. The authors hypothesized that hypoxia may trigger activation of beta-adrenergic receptors on epicardial adipocytes, and this stimulation was prevented by treatment with the non-selective beta blocker metoprolol. This hypothesis is supported by experiments showing that isoproterenol, a non-selective beta-adrenergic agonist, was previously noted to stimulate IL6 and TNFα production in 3T3L1 adipocytes (42). Furthermore, exposing adipocytes to hypoxia *in vitro* results in decreased adiponectin secretion and increased β1 and β2 adrenergic receptor expression (43).

Wang et al. studied the atria and EAT of subjects with (n=28) and without AF (n=36) having coronary artery bypass surgery (34). They found that YKL-40(CHI3L1) mRNA and protein was significantly higher in the EAT of subjects with AF than of those without. There was no difference in the serum levels of YKL-40. There was a positive association between YKL-40 expression in EAT and the collagen fraction of the atrial myocardium. Obesity was an independent risk factor for YKL-40 expression in EAT (34). YKL-40 is a secreted glycoprotein highly expressed in neutrophils, activated macrophages, and other cell types. YKL-40 may act in fibroblast proliferation and matrix deposition, and it’s expression by macrophages in adipose tissue inhibits type I collagen breakdown (44). Interestingly, the expression of YKL-40 is increased in the visceral fat of obese patients and decreases with weight loss (45).

Recently, a provocative study by Shaihov-Taper et al. showed that EAT also releases extracellular vesicles (EVs) (46). EVs are membrane bound vesicles released from all cell types, containing a variety of molecules (e.g. proteins, nucleic acids, and lipids), that can transmit a molecular signal from the releasing cell to a recipient cell (47). EAT from subjects with AF secreted a greater number of EVs than EAT from subjects without AF. The EVs from subjects with AF had higher concentrations of inflammatory and pro-fibrotic cytokines, and lower concentrations of IL-10, VEGF, and sFLT-1 (46). Subsequent proteomic analysis revealed that the EVs from those with AF were related to distinct molecular pathways, including cardiomyopathy, apoptosis, angiogenesis, and fibrosis. Furthermore, these enriched EVs triggered fibrosis, angiogenesis, and facilitated re-entry when co-incubated with mesenchymal, endothelial, and pluripotent stem cells *in vitro (* 46).

In summary, fibrosis, and inflammation of EAT is associated with fibrosis of the underlying atrial myocardium. There are many bioactive factors released from EAT that could potentially cause fibrosis of the atria; this causative effect has yet to be clearly demonstrated (**Table 1**). Nonetheless, it is clear that EAT is responsive to changes in metabolic health and could signal this change to neighboring and possibly even remote systems.

**Table 1:** Factors Released from EAT with Possible Paracrine Effects.

Protective Factors	Mechanism	Pathologic Factors	Mechanism
Adiponectin (29)	Downregulated in EAT of subjects with AF	MCP-1, IL6, TNF-α (41, 46)	Profibrotic and proinflammatory
Apelin	May reduce fibrosis by inhibiting TGFβ1 signaling in atrial myofibroblasts (75)	TGF-β1 (76)	Upregulated in the EAT of subjects with AF. Promotes fibrosis and ENdMT.
Omentin (76)	Downregulated in EAT of subjects with AF. May inhibit TGF signaling.	HIF-1α, Angptl2 (35)	Proinflammatory and profibrotic
		MMP2, MMP9 (41)	Remodeling of adipose tissue stroma
		Activin A (32)	Profibrotic
		Resistin (77)	Proinflammatory
		Visfatin	Proinflammatory
		cTGF (78)	Increased in EAT from subjects with AF. Correlates with atrial fibrosis.
		Leptin	
		YKL-40/CHI3LI (34)	Secreted glycoprotein expressed by activated macrophages, neutrophils, and other cells. Correlates with atrial collagen fraction and increased in EAT of AF subjects.

## Implications for treatment and future directions

### Weight loss

With the recognition that EAT is associated with increased cardiovascular risk there have been several studies examining non-pharmacologic interventions that can reduce the amount of epicardial fat. In general, epicardial fat is a marker of visceral fat, and weight loss strategies tend to impact both depots. Diet and bariatric surgery both result in a significant reduction in epicardial fat (48). A meta-analysis by Saco-Ledo et al. included 10 studies and 521 subjects (49). They found that endurance training also resulted in a significant reduction in EAT. It seems that any method of weight loss can result in a decrease in epicardial fat. Moderate exercise and weight loss have known effects on reducing AF burden (18). Whether or not this is effect is dependent on a reduction in EAT, or simply a reduction in weight, is unknown. For example, in an echocardiographic study of subjects before and after bariatric surgery, weight loss was associated with a 30% reduction in visceral fat area and a 14% reduction in EAT thickness (50). However, despite these reductions, left atrial function remained impaired and left atrial volume and pressure increased (50). Therefore there may be a threshold of obesity or exposure to EAT beyond which remodeling of the left atrium is irreversible. More studies are needed to determine if weight loss results in a reduction in EAT thickness and whether this translates into clinically meaningful results.

### GLP-1 agonists/SGLT2 inhibitors

Liraglutide and other glucagon-like peptide-1 (GLP-1) receptor agonists are indicated for the treatment of diabetes mellitus. GLP-1 receptor agonists also have weight loss effects. Treatment of diabetic patients with GLP-1 receptor agonists lowers cardiovascular events (51). The mechanism by which these drugs exert their beneficial effects is not clear. Iacobellis et al. found that treatment with liraglutide for 6 months causes a rapid and significant decrease in the thickness of EAT as measured by echocardiogram (52). 95 subjects with DM2 were randomized to metformin or metformin plus liraglutide 1.8 mg SC daily. Subjects in the liraglutide and metformin group had a 36% reduction in their EAT thickness at 6 months. Interestingly, the GLP-1 receptor is expressed in EAT (52). Others have shown similar albeit less dramatic results on EAT thickness with GLP-1 receptor agonists exenatide and dulaglutide (53, 54). Potential mechanisms by which GLP1 receptor agonists work in EAT include but are not limited to, reduction in fat mass, improved differentiation of pre-adipocytes, reduced lipogenesis, or browning of EAT (55).

Sodium-glucose co-transporter 2 inhibitors (SGLT2i) reduce blood glucose and cause weight loss in diabetic patients. This class of drugs has revolutionized the treatment of heart failure and are now recommended as one of the four pillars of goal directed medical therapy (56). Apart from weight loss and the natriuretic effects of SGLT2 inhibitors, they are thought to have salutary effects on myocardial metabolism, including a shift in fuel utilization from free fatty acids to b-hydroxybutyrate (55). However, SGLT2 inhibitors have also been shown to reduce EAT thickness by up 20% (55). It is thought that these compounds may reduce foster adipocyte differentiation and reduce the secretion of pro-inflammatory cytokines in EAT (57). Whether or not treatment with SLGT2 inhibitors or GLP-1 receptor agonists reduce incident AF is unknown.

### Statins

Systemic inflammation is a characteristic of chronic obesity. It is known that obese patients are at increased risk of AF and heart failure with preserved ejection fraction. Increased epicardial fat thickness and EAT inflammation may contribute to the development of both AF and HFpEF in obese patients (58). HMG-coa reductase inhibitors or “statins” are known to have anti-inflammatory effects separate from their lipid lowering effects. EAT is known to a greater degree of inflammation on than subcutaneous adipose tissue (SAT). This correlates with a higher average contrast attenuation on CT scan in EAT (-89 HU) than SAT (-129 HU) (59). In a study of 420 women who had serial CT scans to measure coronary artery calcification, the use of statins for one year was found to reduce the attenuation of EAT (-89 HU at baseline vs. -94 HU at follow up, p<0.001). There was no change in the attenuation of SAT of the same subjects suggesting that the anti-inflammatory effect was specific to EAT. Furthermore, this effect was independent of changes in EAT volume, total cholesterol, or coronary calcium (59). The same group had previously shown that statin use was also associated with a reduction in EAT volume over time (60). In 145 patients who had serial coronary angiography, atorvastatin showed a greater effect of reducing EAT thickness than simvastatin/ezetimibe(0.47 mm vs. 0.12 mm, p<0.001) (61). Others have shown that statin use is associated with lower EAT thickness and decreased inflammation in patient having cardiac surgery (62). Statins were also shown to have an inhibitory effect on a broad array of inflammatory cytokines released from EAT *in vitro* (62). It appears clear that statins demonstrate salutary anti-inflammatory properties that are beneficial in metabolic disorders such as obesity, and these effects may be mediated by a reduction in EAT thickness or inflammation (58).

### Ablation

Increased EAT thickness has been associated with recurrence of AF after ablation (63). Larger peri atrial EAT volume is also related to the occurrence of embolic stroke after catheter ablation of AF (64). Some studies have failed to find an association between EAT and AF recurrence after catheter ablation, and it may depend on the stage of AF (paroxysmal vs. persistent) (65). Traditionally AF ablation has used an endocardial approach to isolate the pulmonary veins. Recently, a clinical trial evaluated the efficacy of a hybrid procedure utilizing endocardial and epicardial ablation of the posterior left atrial wall (66). Compared to endocardial ablation alone, those who underwent the hybrid procedure had increased primary effectiveness at 12 months. It is not known whether incidental modification of EAT at the time of this procedure plays is instrumental to its efficacy.

### Modulation of ganglionic plexi within EAT

Activation of both the sympathetic and parasympathetic nervous system is thought to play a role in the initiation of atrial fibrillation (67, 68). Stimulation of the pulmonary veins in dogs does not induce AF unless the adjacent ganglionic plexi are also stimulated (69). A clinical trial of botulinum toxin injection into the epicardial fat pad at the time of cardiac surgery showed a reduction in the incidence of both early and late post-operative AF (70). Interestingly, acetylcholine (Ach) has been found to have acute and chronic effects on epicardial adipocytes *in vitro (* 71). In comparison to subcutaneous adipocytes, epicardial adipocytes have greater increases in calcium flux in response to Ach. Ach also induces MCP-1 expression in epicardial, but not subcutaneous adipocytes, and Epicardial adipocytes have increased expression of the g protein linked muscarinic receptors (mAChR2, mAchR3) (71). Finally, chronic treatment of cells with Ach caused increased lipid accumulation in both subcutaneous and epicardial adipocytes (71). Therefore it seems that Ach may stimulate inflammation and lipid accumulation in epicardial adipocytes, an effect that cut putatively be inhibited by botulinum toxin or other methods. In dogs, ablation of ganglionic plexi with a neurotoxin (resiniferatoxin) decreased sympathetic and GP activity and reduced AF inducibility. Resiniferatoxin is a transient receptor potential vanilloid 1 (TRPV1) agonist (72). These studies highlight the potential utility of chemical modification of ganglionic plexi as an adjunct to hybrid or surgical procedures.

### Browning

“Browning” refers to the possibility of inducing UCP-1 expression in white adipose *via* a pharmacologic or alternative intervention. This would cause increased energy expenditure and insulin sensitivity; a concept with great promise for treatment of metabolic disorders and the induction of weight loss. An additional beneficial aspect of brown adipose tissue is that it is relatively resistant to inflammation induced by high fat diet (25). Two early studies in the field detected increased expression of brown adipose tissue associated genes in EAT (23, 73). However, on a histological basis, EAT appears more like white adipose tissue. Nonetheless, the induction of UCP1 in EAT is an intriguing concept to combat the development of cardiovascular disease associated with EAT (74).

## Conclusion

EAT is a unique fat depot with distinct biochemical and metabolic properties. The exact function of EAT in cardiovascular physiology is unknown. The amount of EAT has been shown to correlate with the development of atrial fibrillation and adverse outcomes after atrial fibrillation ablation. As in other fat depots, it is highly likely that EAT interacts with the autonomic nervous system, and this is a topic that merits further investigation. There are many questions that remain to be answered about this fat depot, but the potential for therapeutic opportunities is intriguing.

## Author contributions

BW contributed to writing the manuscript, editing, and approving the final draft. K-VT created the figure, contributed to writing the manuscript, editing, and approving the final draft. TF contributed to writing the manuscript, editing, and approving the final draft. All authors contributed to the article and approved the submitted version.
